# Supplementary figures and images for: Effects of a spore-forming probiotic blend on bowel habits and physical well-being in adults with functional constipation: A randomized, double-blind, placebo-controlled trial
Source: PLoS One. 2026 Apr 24;21(4):e0337019. doi: 10.1371/journal.pone.0337019 (PMC13108732; doi:10.1371/journal.pone.0337019)

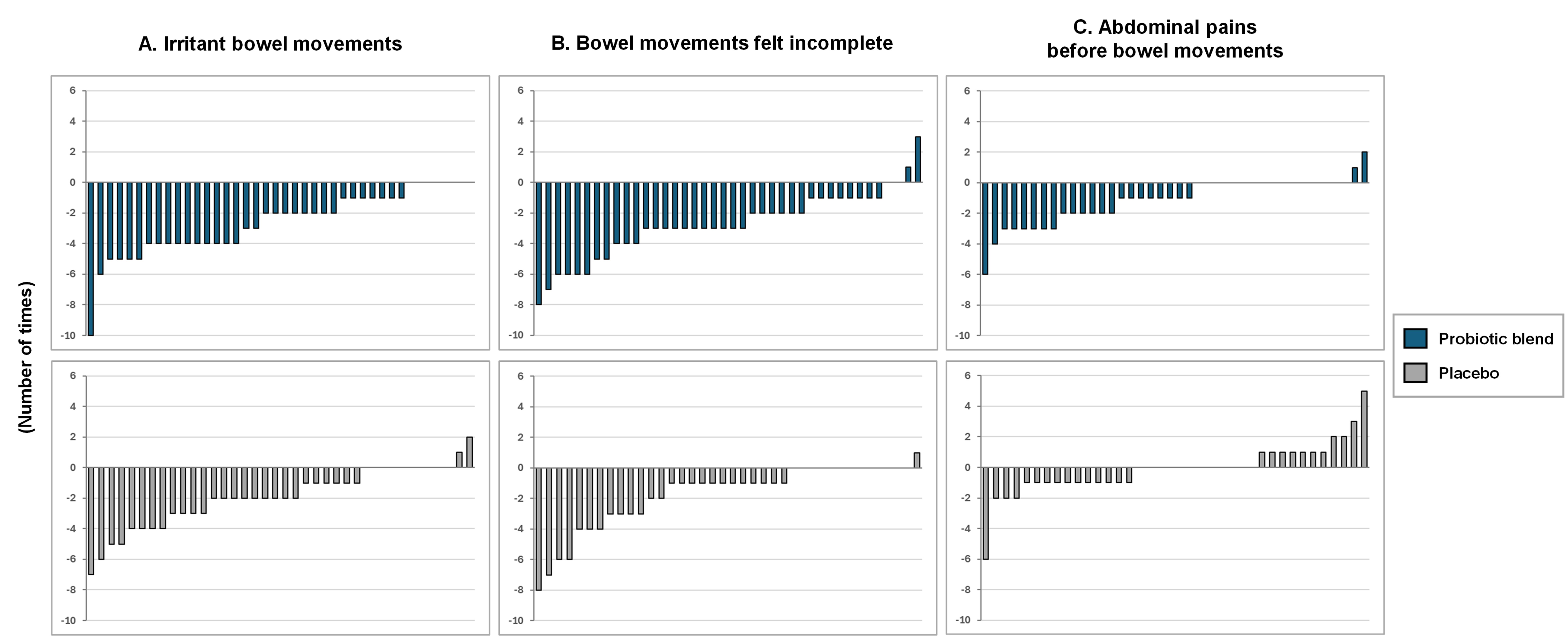

Supplement: S1 Fig — (A) change in number of irritant bowel movements, (B) change in number of incomplete bowel movements felt, and (C) change in number of abdominal pains before bowel movements. The Probiotic blend group is shown in blue, and the placebo group is shown in gray. Outcomes are expressed as the number of times. (JPG) [file pone.0337019.s001.jpg]
