# Supplementary material for: Effects of a spore-forming probiotic blend on bowel habits and physical well-being in adults with functional constipation: A randomized, double-blind, placebo-controlled trial
Source: PLoS One. 2026 Apr 24;21(4):e0337019. doi: 10.1371/journal.pone.0337019 (PMC13108732; doi:10.1371/journal.pone.0337019)
Supplement: S1 Table — (PDF) [file pone.0337019.s002.pdf]

S1 Table. Compositions of the Probiotic Blend and Placebo Products.

Raw material	Probiotic blend (mg)*	Placebo (mg)
Probiotic blend complex	18.0	0.0
Corn starch	209.5	218.5
Maltodextrin	209.0	218.0
silicon dioxide	9.0	9.0
Magnesium stearate	4.5	4.5
Total	450.0	450.0

* Probiotic blend contains 1.0 × 10^9 CFU of probiotic blend complex per capsule (450 mg).
Probiotic blend complex consists of *Weizmannia coagulans* IDCC 1201 (75.8%), *Clostridium butyricum* IDCC 1301 (15.2%), and *Bacillus subtilis* IDCC 1101 (9.0%)
