# Supplementary material for: Effects of a spore-forming probiotic blend on bowel habits and physical well-being in adults with functional constipation: A randomized, double-blind, placebo-controlled trial
Source: PLoS One. 2026 Apr 24;21(4):e0337019. doi: 10.1371/journal.pone.0337019 (PMC13108732; doi:10.1371/journal.pone.0337019)
Supplement: S2 Table — (PDF) [file pone.0337019.s003.pdf]

S2 Table. Daily Intake of Energy and Nutrients.

Variables	Probiotic blend group (n = 40)			Placebo group (n = 38)			*p*
	baseline	4 weeks	change	baseline	4 weeks	change	
Energy (kcal)	1567.66 ± 438.11	1564.09 ± 396.97	-3.56 ± 336.52	1467.01 ± 293.29	1533.90 ± 377.04	66.88 ± 48.60	0.5264
Carbohydrate (g)	223.54 ± 64.43	215.37 ± 56.63	-8.17 ± 47.76	206.11 ± 48.54	205.09 ± 55.85	-1.02 ± 40.17	0.4776
Fat (g)	45.71 ± 19.96	45.91 ± 16.70	0.20 ± 20.99	41.67 ± 13.25	45.53 ± 18.28	3.86 ± 17.90	0.4108
Protein (g)	21.11 ± 8.79	20.89 ± 8.10	-0.22 ± 7.82	21.42 ± 7.05	22.00 ± 9.07	0.58 ± 8.07	0.6576
Dietary fiber (g)	19.60 ± 6.98	18.43 ± 5.39	-1.17 ± 6.04	18.69 ± 5.82	17.89 ± 5.13	-0.76 ± 4.72	0.7608
Water (g)	811.60 ± 328.04	827.10 ± 327.36	15.50 ± 243.34	807.90 ± 294.50	836.20 ± 308.80	28.33 ± 226.80	0.8105
Vitamin A (ug RAE)	411.43 ± 203.71	344.78 ± 119.93	-66.65 ± 226.28	343.50 ± 285.23	320.10 ± 117.80	-23.39 ± 294.20	0.4677
Vitamin D (ug)	1.34 ± 1.05	1.45 ± 0.91	0.11 ± 1.28	1.53 ± 1.43	1.46 ± 1.28	-0.07 ± 1.18	0.5379
Vitamin E (mg)	10.51 ± 3.54	10.53 ± 3.46	0.03 ± 3.78	10.94 ± 3.29	10.10 ± 4.80	-0.84 ± 4.77	0.3781
Vitamin K (ug)	191.31 ± 108.24	160.53 ± 73.61	-30.78 ± 108.25	163.66 ± 120.26	175.68 ± 100.30	12.02 ± 145.40	0.1432
Vitamin C (mg)	67.09 ± 32.34	64.02 ± 34.52	-3.07 ± 38.44	59.62 ± 34.42	61.91 ± 34.52	2.29 ± 37.87	0.5376
Thiamine (mg)	1.14 ± 0.38	1.20 ± 0.49	0.05 ± 0.53	1.01 ± 0.31	1.05 ± 0.28	0.04 ± 0.44	0.9002
Riboflavin (mg)	1.26 ± 0.41	1.34 ± 0.38	0.08 ± 0.43	1.18 ± 0.33	1.22 ± 0.38	0.05 ± 0.49	0.7570
Niacin (mg)	10.66 ± 3.23	11.53 ± 3.91	0.87 ± 4.28	10.59 ± 3.79	11.79 ± 4.58	1.20 ± 5.05	0.7531
Vitamin B6 (mg)	0.46 ± 0.31	0.48 ± 0.27	0.02 ± 0.24	0.43 ± 0.31	0.39 ± 0.24	-0.05 ± 0.30	0.3173
Folate (ug)	241.61 ± 95.24	222.26 ± 62.72	-19.35 ± 75.14	215.25 ± 73.59	217.04 ± 71.97	1.79 ± 74.09	0.2149
Vitamin B12 (ug)	4.10 ± 7.90	3.52 ± 2.69	-0.58 ± 8.42	3.01 ± 2.25	4.07 ± 7.91	1.06 ± 7.90	0.3782
Pantothenic acid (mg)	3.47 ± 1.32	3.40 ± 1.72	-0.07 ± 1.58	3.11 ± 1.34	3.11 ± 1.38	0.00 ± 1.34	0.8413
Biotin (ug)	27.29 ± 16.89	29.26 ± 20.60	1.97 ± 23.27	30.51 ± 24.29	29.74 ± 28.06	-0.77 ± 34.21	0.6827
Calcium (mg)	479.12 ± 188.39	422.22 ± 173.21	-56.89 ± 187.23	437.36 ± 147.68	434.09 ± 161.18	-3.27 ± 206.90	0.2334
Phosphorus (mg)	951.67 ± 269.88	960.08 ± 253.14	8.41 ± 283.00	878.65 ± 214.95	936.13 ± 206.74	57.49 ± 257.00	0.4260
Sodium (mg)	3293.41 ± 1317.96	3436.01 ± 946.13	142.59 ± 1206.40	2852.38 ± 1068.05	3112.78 ± 795.80	260.40 ± 952.70	0.6347
Chlorine (mg)	39.64 ± 154.36	3.87 ± 3.84	-35.77 ± 154.24	7.80 ± 16.55	17.51 ± 76.06	9.71 ± 78.40	0.1036
Potassium (mg)	2330.62 ± 800.34	2226.29 ± 516.33	-104.33 ± 716.28	2109.36 ± 556.00	2131.36 ± 377.77	22.00 ± 533.60	0.3818
Magnesium (mg)	213.66 ± 72.09	202.79 ± 56.74	-10.87 ± 59.38	205.07 ± 57.12	209.15 ± 51.46	4.08 ± 52.45	0.2432
Iron (mg)	10.82 ± 3.39	11.26 ± 3.39	0.44 ± 4.06	9.62 ± 2.10	10.61 ± 3.27	1.00 ± 3.76	0.5333
Zinc (mg)	7.02 ± 2.36	7.47 ± 2.39	0.45 ± 2.06	6.32 ± 2.09	7.22 ± 2.60	0.90 ± 2.95	0.4400
Copper (ug)	594.90 ± 301.01	600.29 ± 334.87	5.39 ± 349.07	632.72 ± 273.60	626.20 ± 345.57	-6.52 ± 353.60	0.8815
Fluorine (mg)	0.00 ± 0.00	0.00 ± 0.00	0.00 ± 0.00	0.00 ± 0.00	0.01 ± 0.01	0.00 ± 0.01	0.2172
Manganese (mg)	2.55 ± 1.17	2.50 ± 1.28	-0.05 ± 0.99	2.67 ± 2.70	2.48 ± 1.09	-0.20 ± 2.24	0.7019
Iodine (ug)	270.53 ± 382.40	231.29 ± 240.78	-39.24 ± 434.91	176.70 ± 250.57	183.99 ± 286.25	7.29 ± 385.20	0.6191
Selenium (ug)	68.78 ± 21.93	73.68 ± 27.87	4.91 ± 25.63	61.76 ± 23.33	67.16 ± 25.18	5.40 ± 29.62	0.9377
Molybdenum (ug)	62.70 ± 30.39	64.93 ± 40.34	2.23 ± 45.29	63.31 ± 32.15	62.23 ± 38.05	-1.08 ± 38.13	0.7286

Values are expressed as means ± standard deviation

Significant difference between baseline and 4 weeks data by paired t test at * < 0.05, ** < 0.01, *** < 0.001.

Significant difference in changes between groups are obtained from independent t-test at * < 0.05, ** < 0.01, *** < 0.001

S2 Table. Daily Intake of Energy and Nutrients. *(continued)*

Variables	Probiotic blend group (n = 40)			Placebo group (n = 38)			*p*
	baseline	4 weeks	change	baseline	4 weeks	change	
Total cholesterol (mg)	214.11 ± 88.40	260.60 ± 144.14	**46.49 ± 126.51***	201.74 ± 102.85	211.02 ± 110.42	9.28 ± 146.20	0.2323
Total fatty acids (g)	37.88 ± 18.60	35.57 ± 12.57	-2.31 ± 20.40	34.32 ± 11.79	36.01 ± 16.98	1.69 ± 17.55	0.3570
Saturated fatty acids (g)	12.98 ± 6.99	11.93 ± 5.11	-1.05 ± 7.65	11.26 ± 4.96	11.75 ± 6.82	0.50 ± 6.52	0.3401
Monounsaturated fatty acids (g)	13.99 ± 8.07	13.09 ± 4.94	-0.91 ± 9.34	12.28 ± 5.19	13.20 ± 7.22	0.92 ± 7.74	0.3513
Polyunsaturated fatty acids (g)	10.54 ± 4.43	10.17 ± 3.89	-0.37 ± 4.80	10.45 ± 3.38	10.76 ± 5.32	0.31 ± 5.06	0.5426
Linoleic acid (g)	8.68 ± 3.83	8.34 ± 3.13	-0.34 ± 4.01	8.21 ± 2.53	8.72 ± 4.40	0.51 ± 4.04	0.3534
Eicosapentaenoic acid (g)	0.12 ± 0.15	0.09 ± 0.11	-0.03 ± 0.16	0.15 ± 0.14	0.12 ± 0.12	-0.03 ± 0.16	0.9926
Docosahexaenoic acid (g)	0.26 ± 0.31	0.20 ± 0.17	-0.06 ± 0.30	0.31 ± 0.31	0.22 ± 0.20	-0.09 ± 0.33	0.6644
Isoleucine (mg)	1706.71 ± 539.05	1841.65 ± 701.13	134.94 ± 622.41	1629.66 ± 543.26	1696.17 ± 550.92	66.51 ± 679.10	0.6437
Leucine (mg)	3130.45 ± 997.20	3348.36 ± 1253.57	217.91 ± 1163.42	2959.36 ± 909.57	3079.84 ± 992.92	120.50 ± 1163.60	0.7127
Lysine (mg)	2426.31 ± 786.33	2654.50 ± 1075.72	228.19 ± 1072.76	2332.24 ± 849.14	2382.78 ± 956.26	50.54 ± 1125.20	0.4775
Methionine (mg)	848.03 ± 277.28	911.44 ± 373.27	63.41 ± 334.70	797.17 ± 294.83	821.86 ± 309.63	24.69 ± 381.70	0.6348
Phenylalanine (mg)	1786.08 ± 552.93	1912.79 ± 670.64	126.71 ± 587.91	1710.49 ± 496.22	1778.68 ± 527.31	68.19 ± 615.40	0.6688
Tyrosine (mg)	1270.66 ± 410.07	1373.22 ± 530.64	102.56 ± 478.06	1221.35 ± 398.74	1259.95 ± 401.48	38.60 ± 510.30	0.5693
Threonine (mg)	1617.39 ± 505.62	1741.74 ± 647.45	124.36 ± 607.75	1551.80 ± 493.92	1589.22 ± 524.87	37.43 ± 638.70	0.5398
Tryptophan (mg)	387.11 ± 116.10	412.68 ± 156.19	25.57 ± 135.03	379.41 ± 109.85	398.31 ± 146.77	18.90 ± 163.70	0.8845
Valine (mg)	2016.72 ± 651.25	2139.51 ± 783.06	122.79 ± 697.11	1892.63 ± 605.72	1975.64 ± 609.24	83.01 ± 754.70	0.8094
Histidine (mg)	1112.86 ± 364.38	1164.74 ± 446.21	51.88 ± 438.24	1059.18 ± 380.66	1114.17 ± 486.90	55.00 ± 539.80	0.9777
