# Supplementary material for: Effects of a spore-forming probiotic blend on bowel habits and physical well-being in adults with functional constipation: A randomized, double-blind, placebo-controlled trial
Source: PLoS One. 2026 Apr 24;21(4):e0337019. doi: 10.1371/journal.pone.0337019 (PMC13108732; doi:10.1371/journal.pone.0337019)
Supplement: S3 Table — (PDF) [file pone.0337019.s004.pdf]

S3 Table. Changes in Physical Activity.

Variables	Probiotic blend group (n = 40)			Placebo group (n =38)			*p-value*[1]
	baseline	4 weeks	change	baseline	4 weeks	change	
Total METs (METs-min/wk)	4343.00 ± 2416.70	**4843.90 ± 2460.40*****	500.94 ± 1329.59	4279.80 ± 3513.80	**4992.70 ± 2453.90****	712.88 ± 1538.00	0.8407
Vigorous (METs-min/wk)	60.00 ± 247.57	76.00 ± 216.60	16.00 ± 315.97	37.89 ± 233.60	101.05 ± 472.26	63.16 ± 247.56	0.7662
Moderate (METs-min/wk)	2373.50 ± 1766.02	**2745.00 ± 1642.31****	371.50 ± 1054.91	2427.37 ± 1815.16	**2971.58 ± 1770.87****	544.21 ± 1214.00	0.7309
Walking (METs-min/wk)	1909.46 ± 1072.56	2022.90 ± 1204.78	113.44 ± 573.71	1814.57 ± 1024.35	1920.08 ± 1132.26	105.51 ± 638.97	0.5824
Sitting (min/wk)	552.00 ± 161.90	538.50 ± 174.70	-13.50 ± 114.28	570.00 ± 146.80	594.50 ± 109.40	24.47 ± 95.85	0.1181

Values are expressed as means ± standard deviation

Significant difference between baseline and 4 weeks data by paired t test at * < 0.05, ** < 0.01, *** < 0.001.

Significant difference in changes between groups are obtained from Mann-Whitney U test at * < 0.05, ** < 0.01, *** < 0.001
