# Supplementary material for: Effects of a spore-forming probiotic blend on bowel habits and physical well-being in adults with functional constipation: A randomized, double-blind, placebo-controlled trial
Source: PLoS One. 2026 Apr 24;21(4):e0337019. doi: 10.1371/journal.pone.0337019 (PMC13108732; doi:10.1371/journal.pone.0337019)
Supplement: S4 Table — (PDF) [file pone.0337019.s005.pdf]

S4 Table. Changes in Bowel Function.

	Probiotic blend group (n = 40)			Placebo group (n = 38)			*p*
	baseline	4 weeks	change	baseline	4 weeks	change	
Number of bowel movements per week	2.63 ± 1.17	**3.63 ± 1.17*****	1.00 ± 0.99	2.66 ± 0.81	**3.61 ± 1.05*****	0.95 ± 0.96	0.6532
Number of times of irritant bowel movements (0-10)	6.03 ± 2.15	**3.40 ± 2.46*****	-2.63 ± 2.13	6.50 ± 2.19	**4.61 ± 2.25*****	-1.89 ± 1.98	**0.0458***
Number of times when bowel movements felt incomplete (0-10)	6.50 ± 2.48	**3.75 ± 2.53*****	-2.75 ± 2.23	6.34 ± 2.28	**4.61 ± 2.54*****	-1.74 ± 2.18	**0.0374***
Number of times of abdominal pains before bowel movements (0-10)	2.93 ± 2.08	**1.80 ± 1.62*****	-1.13 ± 1.54	2.79 ± 2.06	2.68 ± 2.07	-0.11 ± 1.71	**0.0090****
Number of times of abdominal pains during bowel movements (0-10)	2.90 ± 2.33	**1.85 ± 1.76*****	-1.05 ± 1.84	3.18 ± 2.50	**2.66 ± 2.32****	-0.53 ± 1.27	0.0926
Degree of abdominal pain (0-10)	3.63 ± 1.96	**2.38 ± 1.92*****	-1.25 ± 1.53	4.03 ± 2.41	3.32 ± 2.27	-0.71 ± 2.10	0.0778
Amount of gas (0-10)	6.08 ± 2.76	**3.80 ± 2.46*****	-2.28 ± 2.55	5.68 ± 2.57	**3.68 ± 2.34*****	-2.00 ± 2.34	0.2248
Discomfort after bowel movements (0-10)	3.98 ± 2.50	**2.40 ± 2.31*****	-1.58 ± 2.12	4.29 ± 2.60	**2.97 ± 2.51*****	-1.32 ± 2.09	0.3989
Discomfort caused by constipation (0-10)	4.18 ± 2.04	**2.60 ± 2.38*****	-1.58 ± 1.68	5.05 ± 1.87	**3.45 ± 2.21*****	-1.61 ± 1.57	0.2888

Values are expressed as means ± standard deviation

Significant difference between baseline and 4 weeks data by Wilcoxon Signed-Rank Test at * < 0.05, ** < 0.01, *** < 0.001.

Significant difference in changes between groups are obtained from Mann-Whitney U test at * < 0.05, ** < 0.01, *** < 0.001

The difference in changes between groups was calculated after adjustment for disease history and sleep time using analysis of covariance.
